# VISTA drives macrophages towards a pro-tumoral phenotype that promotes cancer cell phagocytosis yet down-regulates T cell responses

**DOI:** 10.1186/s40164-024-00501-x

**Published:** 2024-03-29

**Authors:** Yusheng Lin, Ghizlane Choukrani, Lena Dubbel, Lena Rockstein, Jimena Alvarez Freile, Yuzhu Qi, Valerie Wiersma, Hao Zhang, Karl-Wilhelm Koch, Emanuele Ammatuna, Jan Jacob Schuringa, Tom van Meerten, Gerwin Huls, Edwin Bremer

**Affiliations:** 1 Department of Hematology, University Medical Center Groningen (UMCG), University of Groningen, Groningen, 9713 EZ The Netherlands; 2 Faculty VI, School of Medicine and Health Sciences, Department for human Medicine, Carl von Ossietzky Universität Oldenburg, University Clinic for Gynecology, Oldenburg, Germany; 3 Institute of Precision Cancer Medicine and Pathology, Jinan University Medical College, Guangzhou, Guangdong China; 4 Faculty VI, School of Medicine and Health Sciences, Dept. of Neuroscience, Carl von Ossietzky Universität Oldenburg, Oldenburg, Germany

**Keywords:** VISTA, Phagocytosis, Macrophage biology, Immune checkpoint, Innate immunity

## Abstract

**Background:**

VISTA is a well-known immune checkpoint in T cell biology, but its role in innate immunity is less established. Here, we investigated the role of VISTA on anticancer macrophage immunity, with a focus on phagocytosis, macrophage polarization and concomitant T cell activation.

**Methods:**

Macrophages, differentiated from VISTA overexpressed THP-1 cells and cord blood CD34^+^ cell-derived monocytes, were used in phagocytosis assay using B lymphoma target cells opsonized with Rituximab. PBMC-derived macrophages were used to assess the correlation between phagocytosis and VISTA expression. qRT-PCR, flow cytometry, and enzyme-linked immunosorbent assay were performed to analyze the impact of VISTA on other checkpoints and M1/M2-like macrophage biology. Additionally, flow cytometry was used to assess the frequency of CD14^+^ monocytes expressing VISTA in PBMCs from 65 lymphoma patients and 37 healthy donors.

**Results:**

Ectopic expression of VISTA in the monocytic model cell line THP-1 or in primary monocytes triggered differentiation towards the macrophage lineage, with a marked increase in M2-like macrophage-related gene expression and decrease in M1-like macrophage-related gene expression. VISTA expression in THP-1 and monocyte-derived macrophages strongly downregulated expression of SIRPα, a prominent ‘don’t eat me’ signal, and augmented phagocytic activity of macrophages against cancer cells. Intriguingly, expression of VISTA’s extracellular domain alone sufficed to trigger phagocytosis in ∼ 50% of cell lines, with those cell lines also directly binding to recombinant human VISTA, indicating ligand-dependent and -independent mechanisms. Endogenous VISTA expression was predominantly higher in M2-like macrophages compared to M0- or M1-like macrophages, with a positive correlation observed between VISTA expression in M2c macrophages and their phagocytic activity. VISTA-expressing macrophages demonstrated a unique cytokine profile, characterized by reduced IL-1β and elevated IL-10 secretion. Furthermore, VISTA interacted with MHC-I and downregulated its surface expression, leading to diminished T cell activation. Notably, VISTA surface expression was identified in monocytes from all lymphoma patients but was less prevalent in healthy donors.

**Conclusions:**

Collectively, VISTA expression associates with and drives M2-like activation of macrophages with a high phagocytic capacity yet a decrease in antigen presentation capability to T cells. Therefore, VISTA is a negative immune checkpoint regulator in macrophage-mediated immune suppression.

**Supplementary Information:**

The online version contains supplementary material available at 10.1186/s40164-024-00501-x.

## Introduction

Escape of immune surveillance by cancer cells is a requisite for development of malignancy. To achieve this, cancer cells upregulate immune-inhibitory receptors, while decreasing the expression of immune-costimulatory receptors [1, 2]. Cancer immunotherapies designed to remove these brakes on anticancer T cell activity, so-called immune checkpoint inhibitors (ICIs), have proven remarkably successful. Indeed, ICIs that target the T cell inhibitory receptors CTLA-4 or PD-1/PD-L1 yielded unprecedented clinical responses in end-stage patients and have altered the treatment paradigm for many types of cancer [3, 4].

Another immune checkpoint of particular interest is the V-domain Ig suppressor of T cell activation (VISTA), a type I transmembrane protein that shares similarities with the immunoregulatory molecules PD-1 and CTLA-4 and the costimulatory receptor CD28 [5]. VISTA is encoded by the VSIR gene and is expressed on many cell types within the hematopoietic system. On T cells, VISTA is expressed at relatively low levels yet is upregulated in the hypoxic tumor micro-environment [6], with higher VISTA expression on tumor-infiltrating T cells than peripheral T cells [7]. VISTA has been shown to negatively regulate T cell function, proliferation, and cytokine production [8, 9]. For instance, treatment with a soluble VISTA-Ig fusion protein reduced proliferation and cytokine production in anti-CD3 stimulated CD8 and CD4 T cells [10]. Furthermore, VISTA inhibited antigen-specific T cell activation during interaction with APCs [9] and decreased T cell-mediated IL-10, TNF-α, and IFN-γ production [10, 11]. In a mouse model, VISTA knock-out CD4^+^ T cells responded stronger to antigen stimulation than wildtype cells, whereas treatment with a VISTA-specific agonistic mAb inhibited CD4^+^ T cell activation and proliferation [12]. Further, VISTA expression on ovarian and endometrial cancer cells reduced cytokine production and the number of tumor-infiltrating CD8^+^ T cells in vivo [13].

Interestingly, although known as checkpoint on T cells, the highest expression levels of VISTA are found in myeloid cells, such as monocytes, macrophages, neutrophils, and dendritic cells (DCs) [2, 9]. VISTA has been shown to regulate macrophage and DC activity in various studies. For instance, blockade of VISTA with a monoclonal antibody or absence of VISTA in a Vsir^−/−^ mouse model in combination with a TLR-agonistic vaccine eliminated the suppressive functions of myeloid-derived suppressor cells (MDSC) and tumorigenic DCs and boosted proinflammatory cytokine production. These effects drove development of a T cell-permissive tumor microenvironment (TME) that supported tumor rejection [14]. Furthermore, treatment with a VISTA agonist significantly drove an anti-inflammatory phenotype in macrophages, enhancing the survival in a mouse model with endotoxin shock [15]. However, the mechanism through which VISTA regulates macrophage activity in the context of cancer immune responses remains to be elucidated.

Here, we investigated VISTA in the context of anticancer macrophage biology in vitro and identified that ectopic expression of VISTA induced M2-like transcriptional and functional alterations, both in the monocytic model cell line THP-1 and cord-blood stem cell-derived monocytes. Importantly, the ectopic expression of VISTA strongly reduced the surface levels of the phagocytosis checkpoint SIRPα and, in line with this, augmented basal levels of phagocytosis of a panel of B cell lymphoma cell lines. Notably, expression of the extracellular domain of VISTA also triggered phagocytosis, but only of cell lines expressing a VISTA binding partner. Thus, VISTA facilitated phagocytosis in a ligand-dependent and -independent manner. On monocyte-derived macrophages, endogenous expression of VISTA was higher in M2-like macrophages than in M0- or M1-like macrophages and positively correlated only with phagocytic activity of M2-like macrophages. Importantly, ectopic expression of VISTA down-regulated expression of MHC class I, with a concomitant decrease in specific T cell response as demonstrated for HLA-A2 restricted Human Papilloma Virus E7 TCR-specific T cells. Thus, VISTA potentiated M2 macrophage effector functions, but reduced activation of T cell immunity.

## Results

### Ectopic expression of VISTA drives M2-like macrophage polarization

**Fig. 1:**
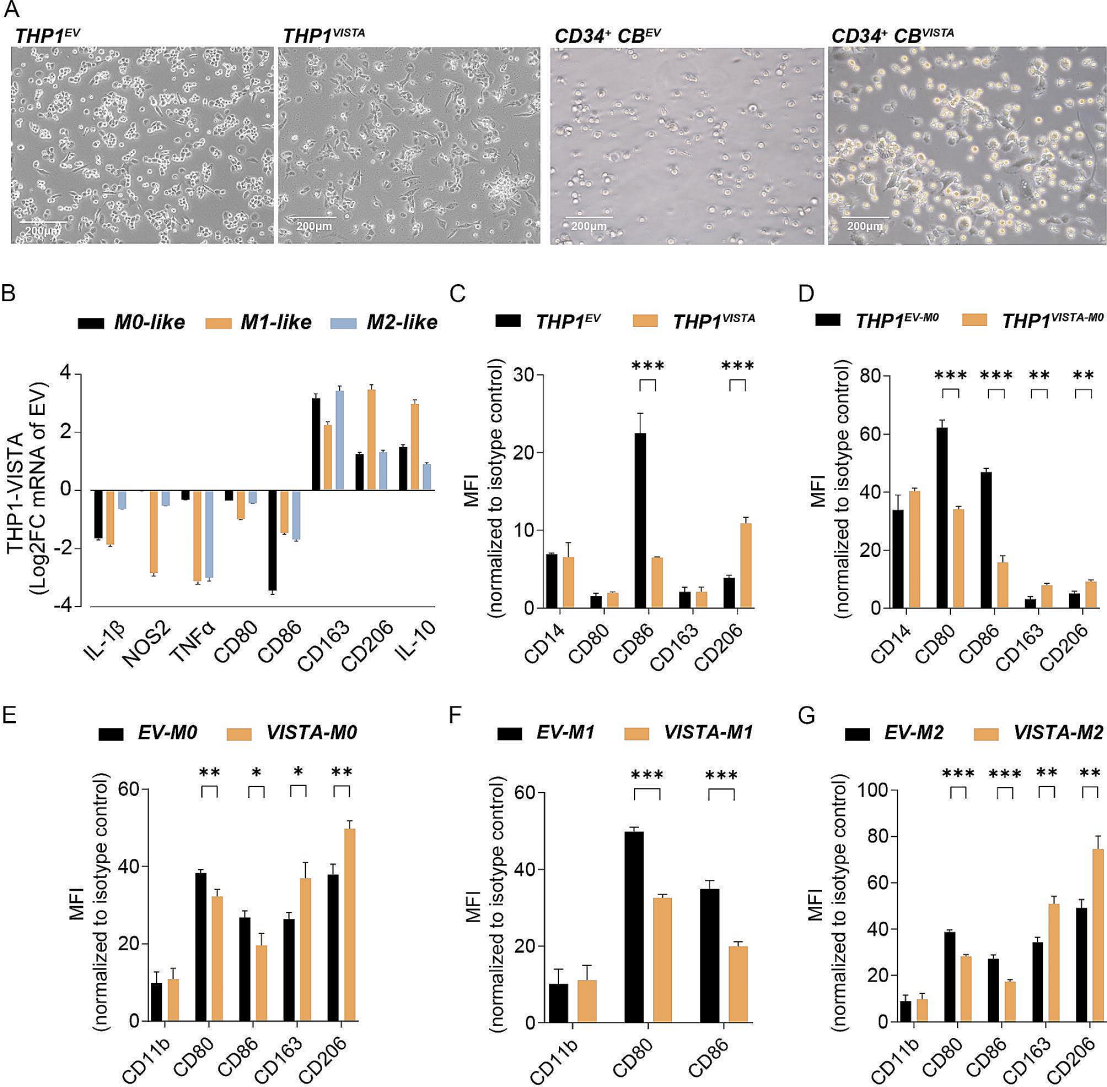
VISTA facilitated macrophage differentiation towards M2-like phenotype. (**A**) Representative pictures of VISTA transduced THP-1 cells upon PMA treatment for 48 h and VISTA transduced CD34^+^ cord blood (CB) cells upon GM-CSF treatment for 7 days. **(B)** The expression of genes was determined by RT-qPCR (relative to control) in THP-1 derived macrophages transduced with VISTA or empty vector (EV). Data is shown as changes in expression between the two groups quantified by the log fold change (FC) from three separate experiments. **(C)** Expression of CD14, CD80, CD86, CD163, and CD206 in VISTA or EV transduced THP-1 cells. **(D)** Expression level of the indicated genes in VISTA or EV transduced THP-1 cells upon 72-hour PMA treatment. **E-G.** Expression level of the indicated genes in M0-like (E), M2-like (F), and M1-like (G) macrophages derived from VISTA or EV transduced CD34^+^ CB cells. ^*^P < 0.05, ^**^P < 0.01, and ^***^P < 0.001 by student’s t-test

Based on literature, we postulated that VISTA would regulate monocyte/macrophage biology and impact on anticancer immunity. To investigate this, VISTA was ectopically expressed in the monocytic cell line THP-1, yielding THP-1^VISTA^, or CD34^+^ cord blood stem cells (CD34^+^ CB), yielding CD34^+^ CB^VISTA^ (Supplementary Fig. 1A). This ectopic expression triggered morphological changes, with enhanced adhesion and spreading, indicative of macrophage differentiation when compared to empty vector control cells (THP-1^EV^ or CD34^+^ CB^EV^) upon treatment with PMA for THP-1 cells or M-CSF for CD34^+^ CB cells (Fig. 1A). These morphological changes in polarized THP-1^VISTA^ cells were accompanied by a significant increase in mRNA levels of pro-tumoral M2-related genes such as CD163, CD206, and IL-10 (Fig. 1B) compared to polarized THP-1^EV^ cells. Reversely, mRNA expression of proinflammatory M1-related genes IL-1β, NOS2, TNFα, CD80, and CD86 was significantly lowered in polarized THP-1^VISTA^ cells (Fig. 1B). In line with this, surface expression of CD206 increased in non-polarized THP-1^VISTA^ cells, whereas CD86 expression was reduced (Fig. 1C). Polarization with PMA treatment further elevated CD206 as well as CD163 expression in THP-1^VISTA^ cells compared to THP-1^EV^ cells, whereas CD86 as well as CD80 expression decreased (Fig. 1D). Correspondingly, ectopic VISTA increased CD206 and CD163 and decreased CD86 and CD80 in (unpolarized/resting) M0-like (Fig. 1E), interferon-gamma (IFN-γ) and lipopolysaccharide (LPS)-polarized M1-like (Fig. 1F), and interleukin-10 (IL-10)-polarized M2c-like (Fig. 1G) macrophages derived from CD34^+^ CB cells. Thus, VISTA promoted polarization of macrophages towards an M2-like phenotype.

### VISTA downregulated the phagocyte-inhibitory ligand SIRP-α and potentiated macrophage-mediated phagocytosis of B cell lymphoma cells

**Fig. 2:**
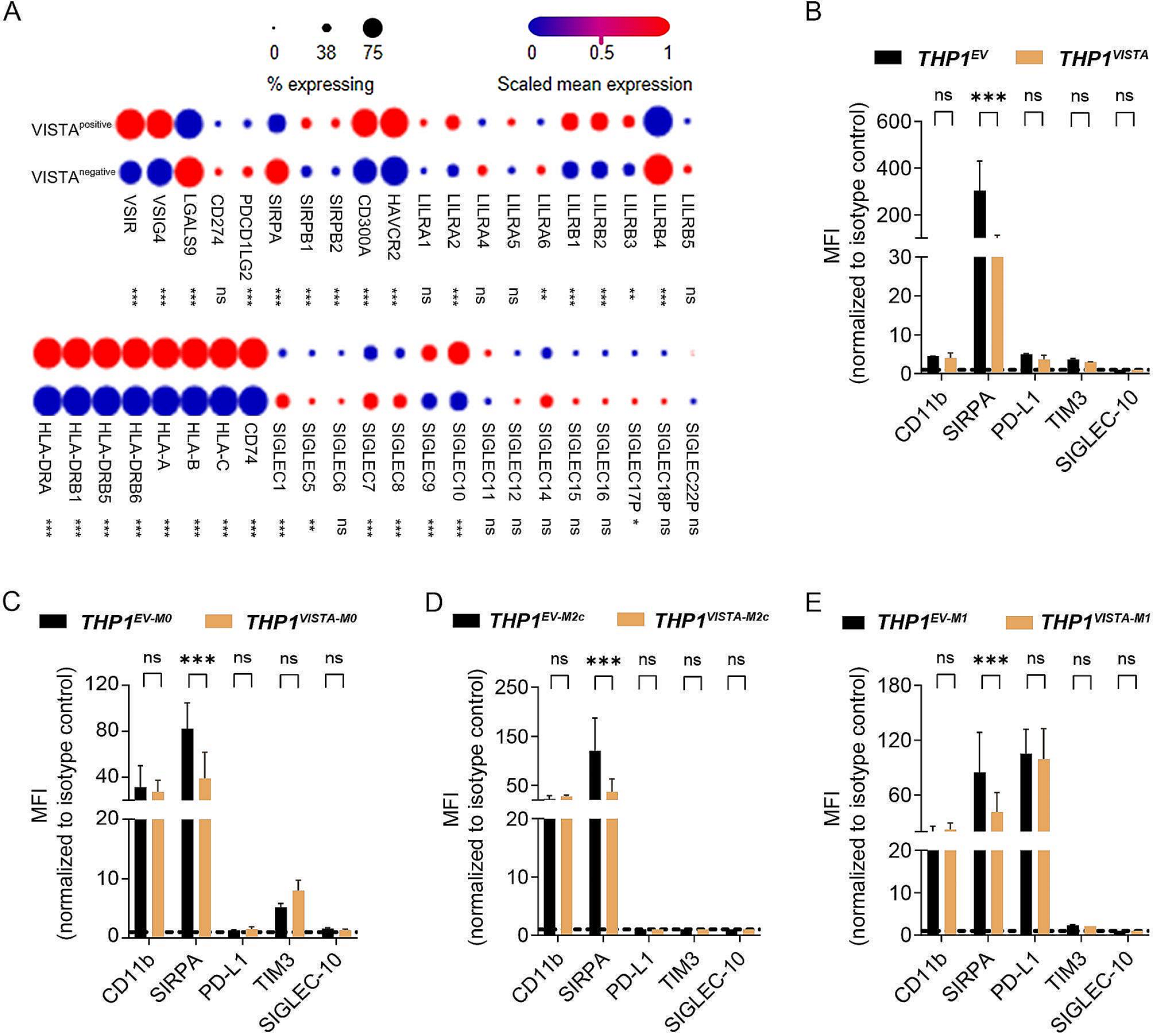
VISTA elicited transcriptional and surface-expression changes of immune checkpoints. (**A**) Dot plot with percentage of cells in VSIR-negative or VSIR-positive tumor-associated macrophages (TAMs) expressing immune checkpoints and normalized expression levels for these immune checkpoints within the two TAM groups. n.s., not significant. ^*^P < 0.05, ^**^P < 0.01, and ^***^P < 0.001 by two-sided Wilcoxon rank-sum test. **(B)** Expression level of the indicated genes in VISTA or EV transduced THP-1 cells. **C-E.** Expression level of the indicated genes in M0-like (C), M2-like (D), and M1-like (E) macrophages derived from VISTA or EV transduced CD34^+^ CB cells. n.s., not significant. ^***^P < 0.001 by student’s t-test

**Fig. 3:**
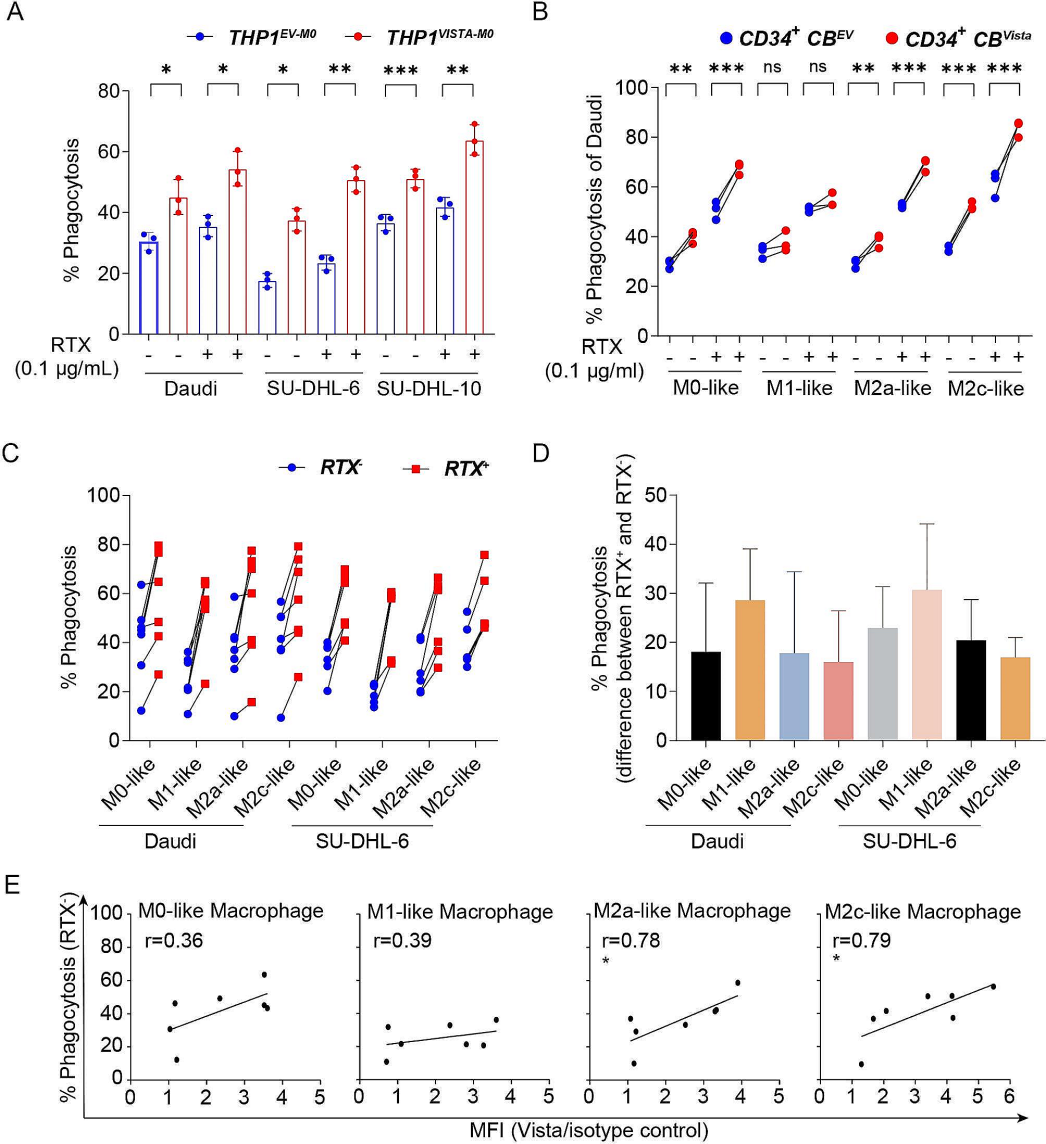
VISTA augmented phagocytosis of cancer cells. (**A**) Quantification of phagocytosis with THP-1 derived M0-like macrophages induced by Rituximab (RTX, 0.1 µg/ml). **(B)** Quantification of phagocytosis with CD34^+^ CB cell-derived M0-like, M1-like, M2a-like, and M2c-like macrophages induced by RTX (0.1 µg/ml). n.s., not significant. ^*^P < 0.05, ^**^P < 0.01, and ^***^P < 0.001 by one-way ANOVA with post hoc intergroup comparisons using Tukey’s test. **(C)** Quantification of phagocytosis with PBMC derived M0-like, M1-like, M2a-like, and M2c-like macrophages induced by RTX (0.1 µg/ml). **(D)** The increase in phagocytosis of PBMC derived macrophages upon RTX (0.1 µg/ml) treatment. **(E)** Correlation between VISTA expression on PBMC derived macrophages and basal levels of phagocytosis. Pearson’s r and linear regression *p* values were shown, ^*^P < 0.05

To further delineate the potential impact of VISTA on macrophage immunity, a public single cell RNA sequencing dataset of tumor associated macrophages (TAMs) was interrogated for expression of immunoregulatory proteins in VISTA^positive^ and VISTA^negative^ TAMs [16]. Interestingly, multiple immune checkpoint genes were differentially expressed between VISTA^positive^ and VISTA^negative^ TAMs (Fig. 2A), with most notably mRNA expression of SIRPα being strongly heightened in VISTA^negative^ TAMs (*p* < 0.001, two-sided Wilcoxon rank-sum test; Fig. 2A). Consistent with these findings, ectopic expression of VISTA downregulated SIRPα in undifferentiated THP-1 cells as well as M0-like, M2c-like, and M1-like THP-1 macrophages (Fig. 2B-E). In line with the downregulation of SIRP-α, phagocytosis of lymphoma cell lines Daudi, SU-DHL-6 and SU-DHL-10 was significantly augmented in M0-like THP-1^VISTA^ compared to THP-1^EV^ (Fig. 3A). The representative FACS results were shown in Supplementary Fig. 2. Ectopic expression of VISTA on CD34^+^ CB-derived macrophages increased phagocytosis in M0 as well as M2a and M2c polarized cells, but not in M1 polarized cells (Fig. 3B). Notably, phagocytosis of B cell lymphoma cell lines upon treatment with opsonizing antibody Rituximab was also increased in THP-1^VISTA^, but this increase was identical to the increase in basal phagocytosis (Fig. 3A and B). Subsequent assessment with confocal microscopy confirmed that cancer cells were phagocytosed and internalized by macrophages (Supplementary Figs. 3, 4). These data indicate that VISTA altered the phagocytic regulatory balance and potentiated the phagocytic activity of macrophages independent of opsonization. We next focused on the effect of endogenous VISTA expression on the phagocytic capacity of human monocyte-derived macrophages (hMDMs) towards lymphoma cells upon Rituximab treatment. Similarly, phagocytosis of lymphoma cells by hMDMs was augmented by Rituximab treatment (Fig. 3C). This enhancement in phagocytosis was consistent across hMDMs differentiated into various macrophage phenotypes (M0, M1, and M2-like) (Fig. 3D). Interestingly, only the phagocytic capacity of M2a- and M2c-like hMDMs was positively correlated with the level of VISTA expression (Fig. 3E). Thus, the phagocytic capacity of M2-like macrophages associated with VISTA. Furthermore, we investigated the impact of ectopic VISTA expression in macrophages on the phagocytosis of normal B cells. Our results indicate that VISTA in THP-1 cell derived macrophages does not augment the phagocytosis of normal B cells derived from the PBMCs of healthy individuals, suggesting a selective effect of VISTA on malignant versus normal cells (Supplementary Fig. 5).

### VISTA increased phagocytosis in a ligand-dependent and -independent manner

**Fig. 4:**
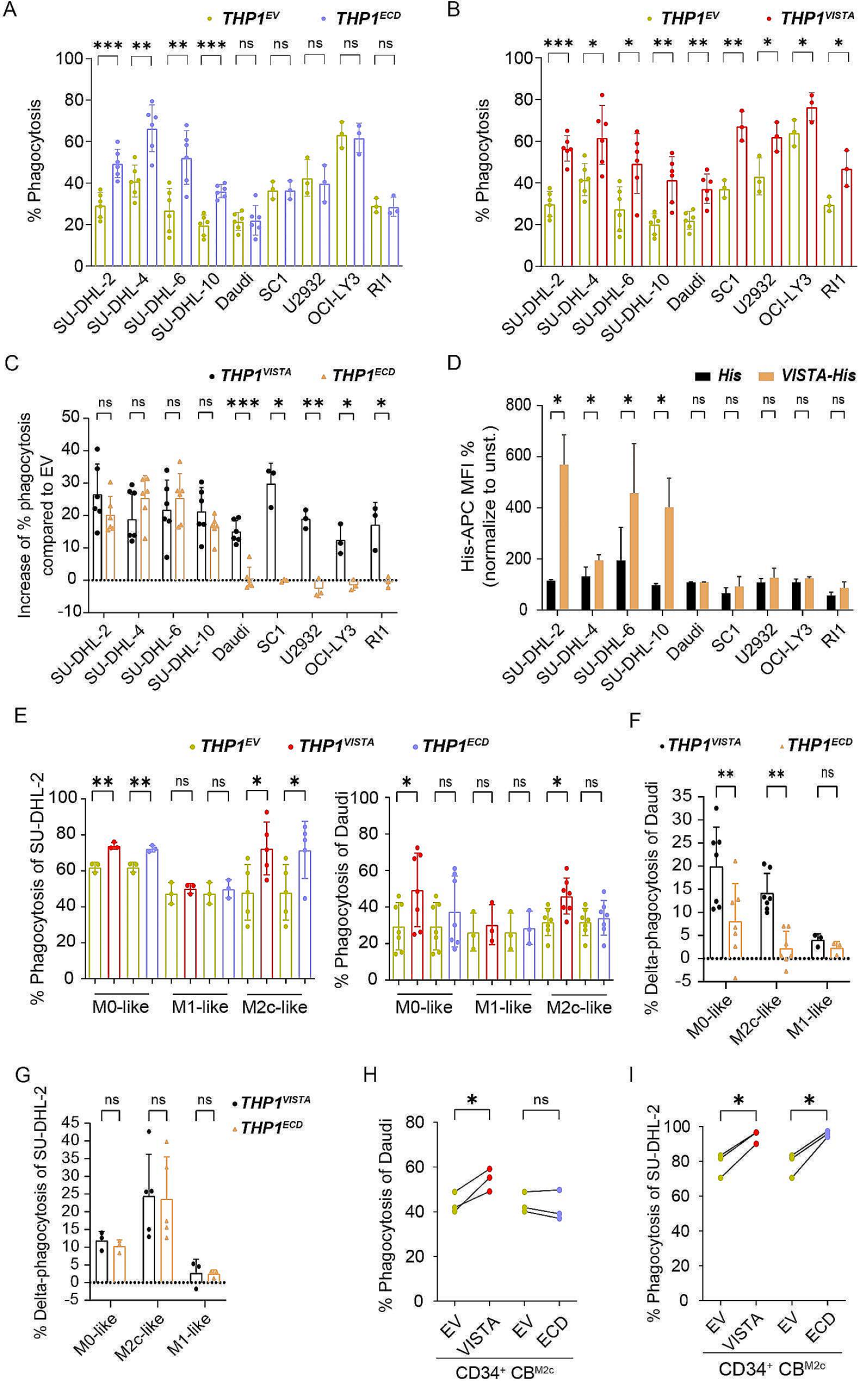
Ectopic expression of VISTA augmented phagocytosis of cancer cells in two manners. (**A**) Phagocytosis of B cell lymphoma cell lines by THP-1 derived M2c macrophages expressing the VISTA extracellular domain (ECD) or empty vector (EV) cells. **(B)** Phagocytosis of B cell lymphoma cell lines by THP-1 derived M2c macrophages expressing VISTA or EV cells. **(C)** Increase of phagocytosis by VISTA and VISTA^ECD^ compared to EV-transduced THP-1 derived M2c macrophages in a panel of DLBCL cell lines. **(D)** Binding of recombinant (His tagged) human VISTA to B cell lymphoma cell lines determined using anti-His tag antibody staining (APC). **(E)** Percentage of phagocytosis of SU-DHL-2 (left panel) and Daudi (right panel) by VISTA-, ECD-, or EV-transduced THP-1 derived macrophages. **F-G.** Percent change in phagocytosis by VISTA- or ECD-transduced THP-1 derived macrophages compared to EV transduced THP-1 derived M0-like, M2-like, and M1-like macrophages in Daudi (F) and SU-DHL-2 (G) cell lines. **H-I.** Percentage of phagocytosis by VISTA-, ECD-, or EV-transduced CD34^+^ CB cell derived M2-like macrophages in Daudi (H) and SU-DHL-2 (I) cell lines. n.s., not significant. ^*^P < 0.05, ^**^P < 0.01, and ^***^P < 0.001 by student’s t-test

To investigate whether VISTA surface interaction or intracellular signaling by VISTA was required for phagocytosis, the effect of surface expression of the extracellular domain (ECD) of VISTA (THP-1^VISTA − ECD^) (Supplementary Fig. 1B) alone on phagocytosis was assessed. Notably, phagocytosis of cell lines SU-DHL-2, SU-DHL-4, SU-DHL-6, and SU-DHL-10 cells was increased in M2 differentiated THP-1^VISTA − ECD^, but phagocytosis of Daudi, SC1, U2932, OCI-LY3, and RL1 cells was not enhanced compared to the control cells THP-1^EV^ (Fig. 4A). In contrast, phagocytosis by M2 differentiated THP-1^VISTA^ was amplified compared to THP-1^EV^ for the complete panel of cell lines, including those not affected by THP-1^VISTA − ECD^ alone (Fig. 4B), with the increase in phagocytosis for the cell lines SU-DHL-2, SU-DHL-4, SU-DHL-6, and SU-DHL-10 comparable to the effect observed for VISTA-ECD (Fig. 4C). This differential impact of VISTA-ECD on phagocytosis in the DLBCL panel suggested that for some cell lines binding of VISTA was sufficient to activate phagocytosis, whereas phagocytosis of other cell lines required VISTA-mediated signaling through the intracellular domain. In line with this, through the utilization of His-tagged recombinant human VISTA (rhVISTA), binding was exclusively identified on SU-DHL-2, SU-DHL-4, SU-DHL-6, and SU-DHL-10 (Fig. 4D). Thus, these cell lines display an interacting partner of VISTA that sufficed to increase phagocytosis by THP-1^VISTA − ECD^. Further experiments using THP-1 derived M0-like, M1-like, and M2-like macrophages and representative lymphoma cell lines (SU-DHL-2 and Daudi) corroborated the ligand-specific effect of VISTA. Notably, only the phagocytosis of SU-DHL-2 was enhanced by VISTA-ECD expression in both M0-like and M2c-like differentiated macrophages, while Daudi cells showed no such increase (Fig. 4E). This ligand-specific enhancement of phagocytosis underscores the importance of the presence of a VISTA receptor on the target cells. Intriguingly, neither VISTA nor VISTA-ECD potentiated phagocytosis of any of the cell lines by M1-like macrophages (Fig. 4E). Additionally, the increase in phagocytosis from baseline (termed delta phagocytosis) of Daudi cells by VISTA was greater than that of VISTA-ECD expressing THP-1 macrophages (Fig. 4F). However, no significant difference in delta phagocytosis was detected in SU-DHL-2 between VISTA and VISTA-ECD expressing THP-1 macrophages (Fig. 4G). Similar to the THP-1 model system, phagocytosis of SU-DHL-2 but not Daudi was enhanced in CD34^+^ CB-derived macrophages upon ectopic expression of VISTA-ECD in M2-like macrophages, whereas expression of full-length VISTA triggered phagocytosis of both Daudi and SU-DHL-2 (Fig. 4H, I). Together, these data suggest a ligand-dependent as well as a cell-intrinsic function of VISTA in promoting phagocytosis by macrophages.

### VISTA triggered anti-inflammatory cytokine secretion and decreased the antigen presentation ability of macrophages

**Fig. 5:**
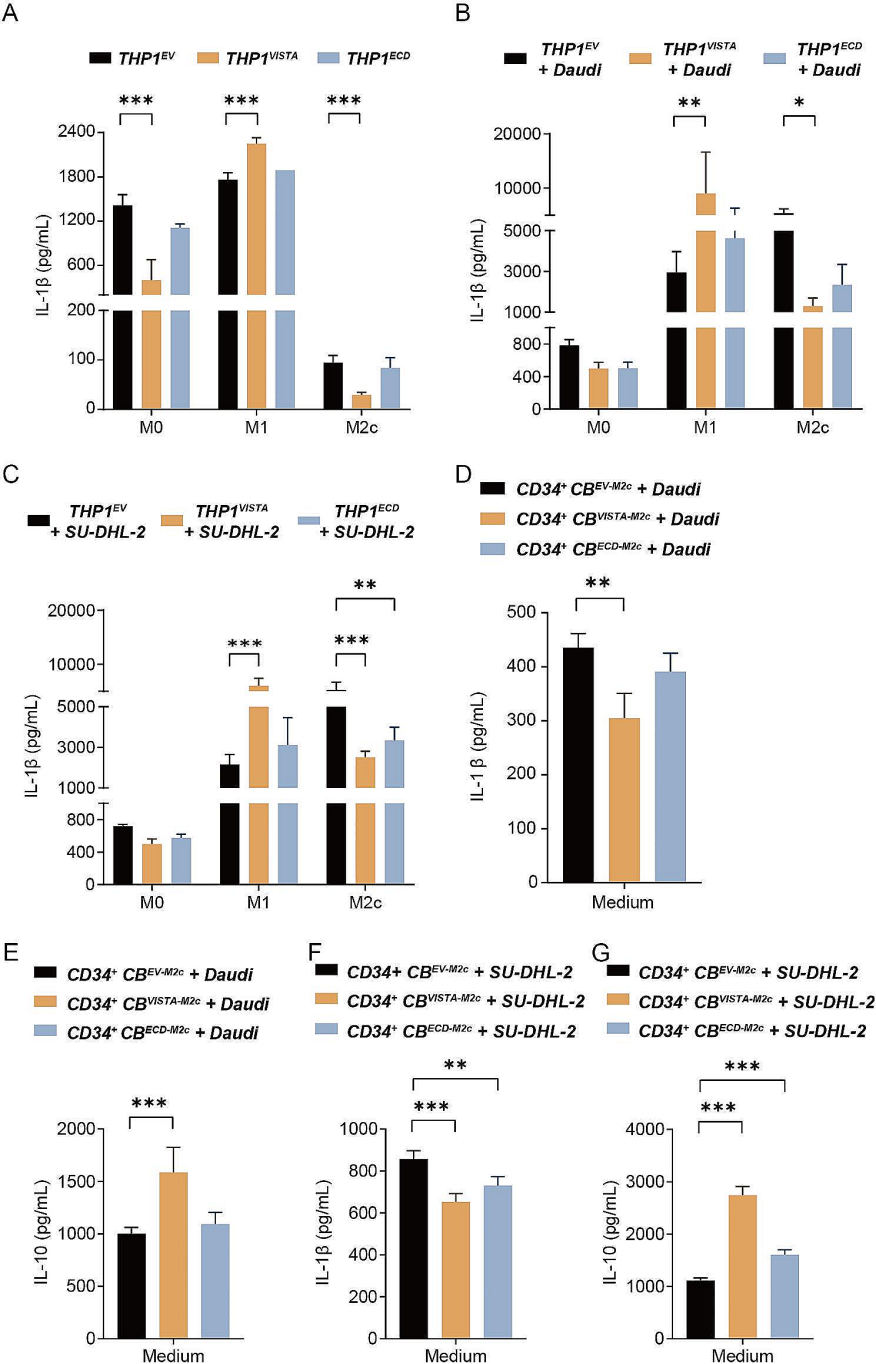
VISTA triggered changes of cytokine secretion during phagocytosis. **A**. Measurement of IL-1β in supernatants from VISTA-, ECD-, or EV-transduced THP-1 derived macrophages. **B-C.** IL-1β concentration in the supernatant of VISTA-, ECD-, or EV-transduced THP-1 derived macrophages after 3 h phagocytosis experiments with Daudi (B) and SU-DHL-2 (C) cells. **D-E.** IL-1β (D) or IL-10 (E) concentration in the supernatant of VISTA-, ECD-, or EV-transduced CD34^+^ CB-derived macrophages after phagocytosis experiment with Daudi cells. **F-G.** IL-1β (F) and IL-10 (G) concentration in the supernatant of VISTA-, ECD-, or EV-transduced CD34^+^ CB-derived macrophages after phagocytosis experiment with SU-DHL-2 cells. ^*^P < 0.05, ^**^P < 0.01, and ^***^P < 0.001 by one-way ANOVA with post hoc intergroup comparisons using Dunnett’s test

Next, the effect of VISTA on cytokine production by macrophages was investigated, with secretion of the pro-inflammatory cytokine IL-1β being decreased in monocultures of M0 and M2-like differentiated THP-1^VISTA^, but increased in M1-like differentiated THP-1^VISTA^ compared to THP-1^EV^ (Fig. 5A). Furthering the investigation into the role of VISTA during phagocytic processes, we conducted a series of 3-hour phagocytosis experiments using Daudi and SU-DHL-2 lymphoma cell lines as targets. Notably, following the engagement with Daudi cells, IL-1β secretion was reduced in the supernatant of M2-like differentiated THP-1^VISTA^ macrophages. A similar decrement in IL-1β secretion was observed in the supernatant following phagocytosis of SU-DHL-2 cells by both M2-like differentiated THP-1^VISTA^ and THP-1^VISTA − ECD^ macrophages (Fig. 5B, C). The influence of VISTA was also evaluated in CD34^+^ CB-derived macrophages. Post a 3-hour phagocytosis challenge with Daudi cells, the presence of VISTA in M2-like macrophages derived from CD34^+^ CB significantly dampened IL-1β secretion while concurrently enhancing the immunoregulatory cytokine IL-10 secretion. (Fig. 5D, E). In the supernatant of M2-like macrophages engaged in phagocytosis of SU-DHL-2 cells, ECD expression had the same effect as VISTA on IL-1β and IL-10 secretion (Fig. 5F, G).

**Fig. 6:**
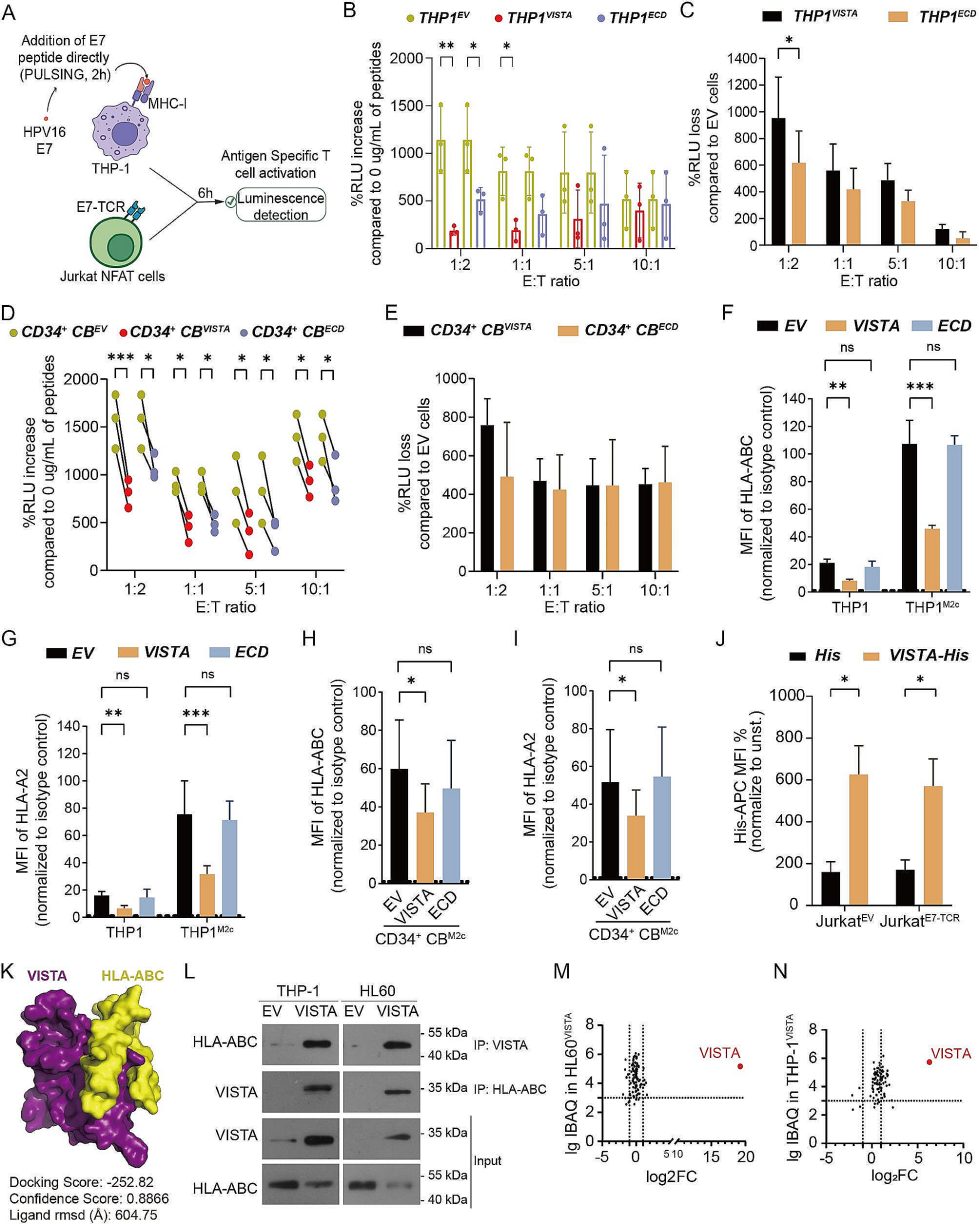
VISTA suppressed antigen-specific TCR activation. (**A**) Schematic illustration of the luminescence assay with antigen-specific TCR Jurkat NFAT-luminescence reporter cells. In brief, E7 TCR Jurkat NFAT cells were co-cultured with THP-1 derived macrophages that were pulsed with HPV-16 E7 (10 µg/mL). Luciferase activity was detected after 6 h in Relative Light Units (RLU). **(B)** RLU after co-culture of Jurkat NFAT cells with THP-1 derived M2-like macrophages at the indicated effector:target (E:T) ratios. Fold increase of normalized RLU activity in Jurkat cells co-cultured with HPV-16 E7 treated cells was calculated over the values from untreated co-cultures. ^*^P < 0.05 and ^**^P < 0.01 by two-way ANOVA with post hoc intergroup comparisons using Tukey’s test. **(C)** RLU loss in Jurkat cells co-cultured with VISTA or ECD transduced THP-1 cells compared to that co-cultured with THP1^EV^ cells. ^*^P < 0.05 by student’s t-test. **(D)** RLU in a co-culture of Jurkat NFAT cells with CD34^+^ CB cell derived M2-like macrophages at the indicated E:T ratios. Fold increase of normalized RLU activity in Jurkat cells co-cultured with HPV-16 E7 treated cells was calculated over the values from that co-cultured with untreated cells. ^*^P < 0.05 and ^***^P < 0.001 by two-way ANOVA with post hoc intergroup comparisons using Tukey’s test. **(E)** RLU loss in Jurkat cells co-cultured with VISTA or ECD transduced CD34^+^ CB cell derived macrophages compared to co-cultures with EV transduced CD34^+^ CB cell derived macrophages. **F-G.** Expression of HLA-ABC (F) and HLA-A2 (G) in VISTA, ECD, or EV transduced THP-1 derived M2-like macrophages. **H-I.** Expression levels of HLA-ABC (H) and HLA-A2 (I) in VISTA-, ECD-, or EV-transduced CD34^+^ CB cell derived M2-like macrophages. **J.** Binding of recombinant (His tagged) human VISTA protein to VISTA- or EV-transduced Jurkat cells measured using APC-conjugated anti-His antibody. **K.** Visual representation of the most optimal molecular binding model between VISTA and HLA-ABC using the HDOCK Server. **L.** Co-IP western blot for VISTA and HLA-ABC in THP-1^EV^, THP-1^VISTA^, HL60^EV^, and HL60^VISTA^ cells. **M.** Log10-transformed IBAQ intensities of quantified membrane proteins identified through mass spectrometry, obtained from anti-HLA-ABC immunoprecipitation (IP) of HL60^VISTA^ cells. The dashed horizontal line represents the Log10 IBAQ threshold of 3. The dotted vertical lines enclose a region where the log2 fold change of protein quantities, obtained from anti-HLA-ABC IP of HL60^VISTA^ cells compared to anti-HLA-ABC IP of HL60^EV^ cells, ranges from − 1 to 1. **N.** Log10-transformed IBAQ intensities of quantified membrane proteins identified through mass spectrometry, obtained from anti-HLA-ABC IP of THP-1^VISTA^ cells. The dashed horizontal line represents the Log10 IBAQ threshold of 3. The dotted vertical lines enclose a region where the log2 fold change of protein quantities, obtained from anti-HLA-ABC IP of THP-1^VISTA^ cells compared to anti-HLA-ABC IP of THP-1^EV^ cells, ranges from − 1 to 1. ^*^P < 0.05, ^**^P < 0.01, and ^***^P < 0.001 by one-way ANOVA with post hoc intergroup comparisons using Tukey’s test

Antigen-presenting cells (APCs) such as macrophages present peptide antigens in the context of Major Histocompatibility Complex (MHC) to T-cells to trigger tumor-specific T cell immunity [17]. To assess the antigen presentation capacity of VISTA-expressing macrophages, Human Papilloma Virus E7 specific T-cell receptor (TCR)-expressing Jurkat luminescence reporter cells were cultured with E7 peptide pulsed THP-1-derived M2-like macrophages (Fig. 6A). The luminescence readout served as a quantitative measure of TCR signaling, and hence, the efficiency of antigen presentation by the macrophages. Interestingly, although THP-1^VISTA^ and THP-1^ECD^ had enhanced phagocytic capability compared to THP-1^EV^, E7 TCR signaling induced by THP-1^VISTA^ and THP-1^ECD^ was significantly lower compared to THP-1^EV^ in response to the E7 peptide (Fig. 6B). In addition, the reduction of E7 TCR signaling for the Jurkat cell line responding to THP-1^ECD^ was equal to THP-1^VISTA^ compared with that responding to THP-1^EV^ except the cells in 1:2 E:T ratio (Fig. 6C), suggesting that the presence of VISTA, regardless of whether it is the full-length protein or just its extracellular domain, negatively impacts the TCR signaling process. A similar decrement in the antigen presentation capability was observed in CD34^+^ CB-derived M2-like macrophages upon VISTA expression (Fig. 6D), also with no difference in the decrease of E7 TCR signaling for the Jurkat cell line responding to THP-1^ECD^ and THP-1^VISTA^ compared with that responding to THP-1^EV^ (Fig. 6E). Intriguingly, the ectopic expression of VISTA but not ECD in THP-1 and CD34^+^ CB-derived M2-like macrophages downregulated HLA-ABC and specifically HLA-A2 levels, the key determinant in antigen presentation to CD8^+^ T Cells (Fig. 6F-I). These results suggest that a direct interaction between MHC-I and VISTA via the ECD as well as signal transduction through the intracellular domain of VISTA might be involved in antigen presentation. Moreover, recombinant human rhVISTA was shown to directly bind to Jurkat cells (Fig. 6J), indicating that an interacting partner of VISTA in Jurkat cells might also influence activation in response to THP-1^VISTA^ or THP-1^VISTA − ECD^ cells. This observation hints at a complex interplay between VISTA, its ligands, and the TCR signaling pathway, potentially influencing T cell activation and the subsequent immune response against tumors.

To identify potential direct interaction partners of VISTA on macrophages, an effective approach using GFP-tagged VISTA (VISTA-GFP) for immunoprecipitation followed by mass spectrometry analysis (IP-MS) was employed. The choice of HL60 cells as the model system for expressing VISTA-GFP, as opposed to THP-1 cells which naturally express minimal levels of VISTA, facilitated a more robust identification process of potential VISTA interactors. (Supplementary Fig. 1C). Out of the 1112 interacting proteins identified, 344 were found to exclusively interact with VISTA-GFP (Supplementary Fig. 6), highlighting the specificity of these interactions. Further analysis leveraging Gene Ontology (GO) term categorization revealed that VISTA-interacting proteins were significantly placed in functional categories involved in antigen presentation/processing, such as protein transport, T cell receptor signaling, and antigen-receptor mediated signaling (Supplementary Fig. 6). Upon subsequent manual inclusion and annotation of cell surface proteins, using the published Surfaceome dataset, 23 surface proteins of interest were identified (Supplementary Fig. 6). Among these were various molecules of the HLA class I and class II family, underscoring the potential impact of VISTA interactions on the antigen presentation machinery of macrophages (Supplementary Fig. 6). To elucidate the direct interaction between VISTA and HLA-ABC, we undertook an extensive analysis of protein-protein docking using the HDOCK server (http://hdock.phys.hust.edu.cn/). This investigation unveiled a strong likelihood of interactions occurring between VISTA and HLA-ABC, supported by a high confidence score of 0.8866 within the range of 0 to 1 (Fig. 6K and Supplementary Table 1). Confirmatory antibody-based IP using an anti-VISTA antibody-based further validated that VISTA and HLA-ABC were co-immunoprecipitated (Fig. 6K). Further, upon reverse IP using an anti-HLA-ABC antibody, VISTA was co-IPed in VISTA-expressing cell lines (Fig. 6L). Similarly, mass spectrometry on the anti-HLA-ABC antibody-based IP from THP-1^EV^, THP-1^VISTA^, HL60^EV^, and HL60^VISTA^ cells yielded VISTA as a prominent hit in HL60^VISTA^ and THP-1^VISTA^ (Fig. 6M-N). Taken together, although VISTA expression had a positive impact on tumor cell phagocytosis by macrophages, the dampened pro-inflammatory cytokine secretion and reduction in antigen presentation by VISTA^+^ macrophages hindered further adaptive immune responses, yielding a suppressive anti-tumor immune signature of VISTA.

### VISTA is expressed on all monocytes and T cells of lymphoma patients, but is not detected on a subset of healthy subjects

**Fig. 7:**
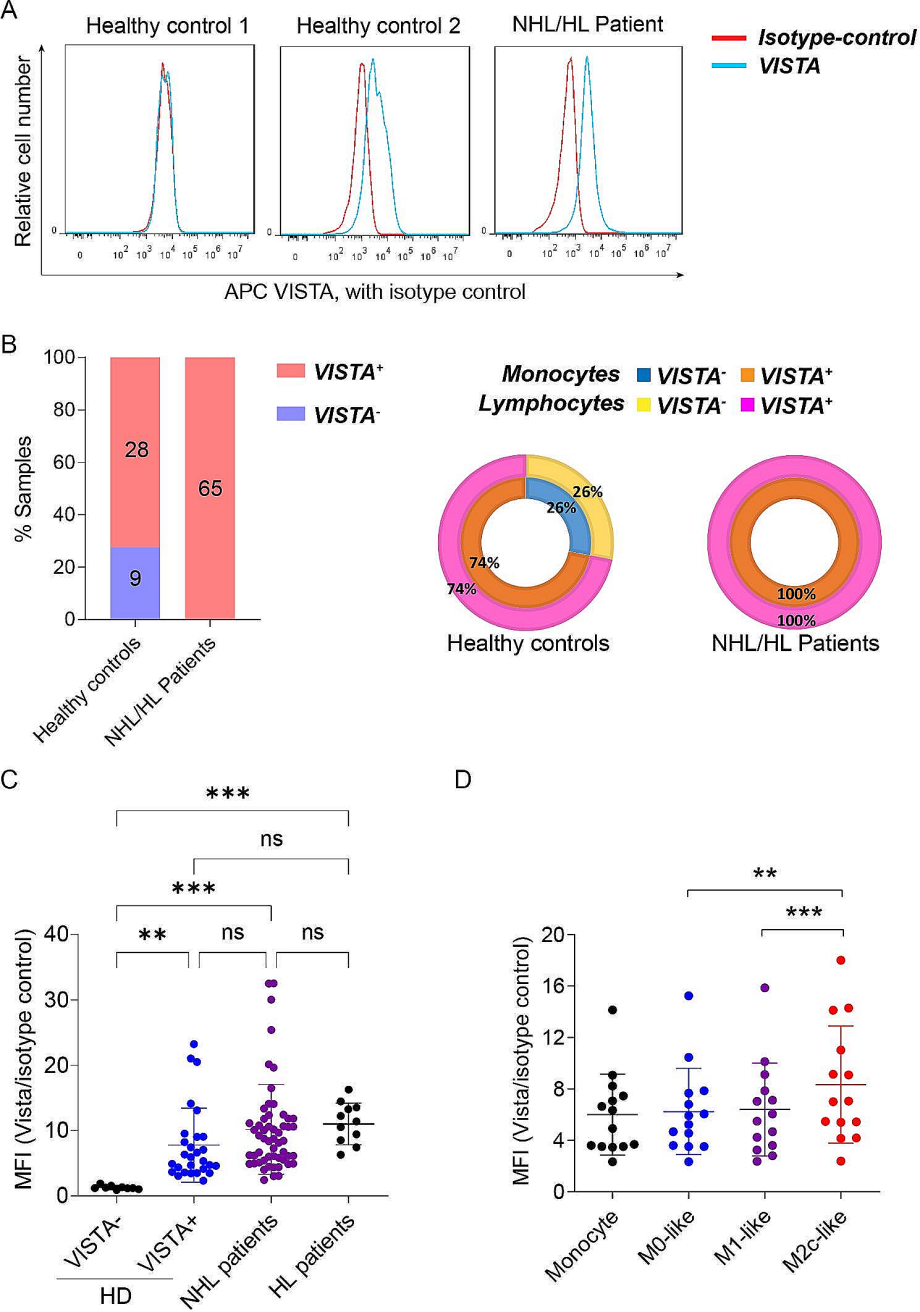
VISTA surface expression on monocytes/macrophages and lymphocytes. (**A**) Representative flow cytometry diagram demonstrating surface expression of VISTA on CD14^+^ monocytes from non-Hodgkin or Hodgkin lymphoma patient PBMCs and healthy PBMCs. **(B)** Comparison of VISTA expression on CD14^+^ monocytes from non-Hodgkin or Hodgkin lymphoma (NHL/HL) patient PBMCs and healthy PBMCs (left panel). Sunburst plots of percent of donors with VISTA expression on CD14^+^ monocytes (inner ring) and CD3^+^ lymphocytes (outer ring) in NHL/HL patient PBMCs and healthy PBMCs (right panel). **(C)** Expression level of VISTA on CD14^+^ monocytes from NHL/HL patient PBMCs and healthy PBMCs. **(D)** Expression level of VISTA on VISTA^+^ CD14^+^ monocytes and M0-like, M1-like, and M2-like macrophages. ^**^P < 0.01 and ^***^P < 0.001 by one-way ANOVA with post hoc intergroup comparisons using Tukey’s test

When evaluating endogenous expression of VISTA on monocytes from blood of 65 lymphoma patients (54 non-Hodgkin and 11 Hodgkin) all of the samples uniformly expressed VISTA (Fig. 7A and B). Contrastingly, monocytes of only 28 of 37 healthy donors (HD) expressed VISTA, with the remaining 9 HD (∼ 24%) lacking surface expression of VISTA (Fig. 7A and B). Of note, the level of VISTA expression, as determined by fluorescent intensity, was comparable in lymphoma patients and healthy donors for the VISTA-positive HD samples (Fig. 7C). Further analysis revealed a correlation between VISTA expression on monocytes and CD3^+^ T cells within the same individuals. Specifically, those HD lacking VISTA on monocytic cells also did not express VISTA on CD3^+^ T cells, whereas CD3^+^ T cells of all lymphoma patients also expressed VISTA (Fig. 7B), pointing to a ubiquitous presence of VISTA in the lymphoma immune microenvironment and hinting at its importance in lymphoma immunobiology. An intriguing aspect of VISTA expression was observed when examining macrophages derived from VISTA-positive monocytes. All such macrophages retained expression of VISTA post differentiation, with expression on M2c-like macrophages being significantly higher than on M0 or M1-like macrophages (Fig. 7D). This differential expression profile underscores the potential for VISTA to influence macrophage polarization, possibly steering immune responses towards a more anti-inflammatory phenotype in the context of lymphoma.

## Discussion

VISTA has previously been established as an immune checkpoint that inhibits T cell proliferation and activation. Here, we demonstrate that in innate immunity the expression of VISTA drives macrophages towards M2-like phenotype and decreases the expression of the key phagocytic protein SIRPα. In line with this, ectopic expression of VISTA potentiated phagocytosis, while endogenous VISTA on M2 macrophages positively correlated with phagocytosis of cancer cells. VISTA-driven phagocytosis was associated with a marked decrease in pro-inflammatory cytokines and diminished TCR-mediated activation of T cells. Therefore, our findings confirm VISTA as a negative immune checkpoint for macrophage-mediated anticancer immunity.

The presence of so-called M2 tumor-associated macrophages (M2-TAM) in the tumor microenvironment associates with poor clinical outcome and resistance to cancer therapy [18–20]. In our study, we identified that ectopic expression of VISTA drives monocytes towards an M2 macrophage phenotype, indicating that VISTA may promote M2 TAM differentiation. This is in line with a previous study where VISTA expression has been correlated with amplified CD68^+^ tumor-associated macrophages in pancreatic tumors resulting in poor clinical outcomes [21]. Furthermore, VISTA deficient macrophages had a reduced chemotaxis and migration to the tumor microenvironment [22]. VISTA thus appears to alter macrophage biology in a similar way to other types of checkpoints, such as LILRB2, CD163, and CD200 [23–25]. Of particular interest, prior studies have documented the continuous release of soluble VISTA by human peripheral monocytes, with this release modulated by various pro- and anti-inflammatory signals that subsequently impact macrophage polarization [26]. Notably, activated M1 macrophages generally exhibit a higher release of VISTA compared to M2 macrophages [26]. This discovery implies that the observed lower VISTA in M1-like macrophages, in comparison to M2-like macrophages in our study, might be due to elevated release of VISTA within the M1-like macrophages.

Therapies targeting such innate checkpoints have yielded significant antitumoral activity by promoting the polarization of macrophages towards anti-tumor M1 phenotype, an effect that might be achieved with VISTA antagonism as well. VISTA expression in THP-1 cell lines and patient-derived macrophages enhanced phagocytosis of cancer cells mainly by M2 macrophages, with endogenous VISTA only correlating with phagocytosis in M2 macrophages. Of note, anti-CD47 therapies promote M1 polarization and phagocytosis of cancer cells by both M1 and M2 macrophages, with more prominent phagocytosis by M1 than M2 [27], reversely to what we observed here with VISTA. Furthermore, a decreased IL-1β and increased anti-inflammatory cytokine IL-10 levels were also detected after phagocytosis of cancer cells by VISTA^+^ M2 macrophages. This pattern of cytokine secretion from VISTA^+^ macrophages is similar to that of TAMs, where the secretion of anti-inflammatory cytokines results in an immunosuppressive microenvironment and enhances tumor progression [28, 29]. Of note, M2-like TAMs have poor antigen presentation capacity and suppress T cell activation and proliferation through secretion of IL-10 and TGF-β [30–32]. Thus, in the case of VISTA, the enhanced phagocytosis of cancer cells by M2 macrophages may serve to suppress adaptive immune activation since phagocytosis is accompanied by secretion of anti-inflammatory cytokines.

In line with the anti-inflammatory cytokine secretion, VISTA^+^ macrophages in our study had a downregulated surface expression of HLA-ABC and were less effective at activating HPV E7 TCR-specific T cell responses. This is in line with another study in which VISTA on APCs had a negative impact on CD4^+^ T cell responses by suppressing antigen-specific TCR activation [9]. However, the mechanism by which VISTA inhibits T cell activation through macrophages is not yet clear. One important finding in this regard is the downregulation of important surface proteins that regulate antigen presentation (i.e., HLA-ABC) on VISTA^+^ macrophages. Additionally, we found that both MHC-I (e.g., HLA-B) and MHC-II molecules (HLA-DRA, HLA-DRB, and HLA-DQA) appear to be direct interacting partners of VISTA using immunoprecipitation-mass spectrometry (IP-MS), although confirmatory experiments are still ongoing to validate this finding. Nevertheless, this finding suggests that VISTA may also directly interfere with both CD8^+^ cytotoxic and CD4^+^ helper TCR/MHC interaction.

As has been previously reported, the high therapeutic efficiency of CD47-SIRPα blockade appears to stem from providing a bridge from innate immune activation to more effective T cell priming and activation in preclinical studies [33, 34]. Thus, a clear understanding of the impact of tumor phagocytosis by macrophages is critical. From the above data, it seems worthwhile to explore targeting of the VISTA pathway in innate immunity as a means of preventing MHC downregulation to augment antigen presentation and enhance anti-tumor T cell immunity. In this regard, in several studies the ability of VISTA blocking antibodies to restore T cell immunity and reduce tumor growth was demonstrated [35–37], with the first VISTA antagonist already being clinically evaluated (NCT02812875). It would be of interest to assess innate immune markers during treatment with VISTA antagonist.

Interestingly, all patient-derived monocytes evaluated in this study were positive for VISTA at the mRNA and protein level, whereas approximately a third of the healthy subjects had very low mRNA expression and no detectable surface VISTA. This difference in VISTA expression between patients and healthy subjects is intriguing and was also observed for VISTA expression in T cells. The underlying reason for this differential expression is yet to be determined, but could not be attributed to epigenetic suppressive marks, as these were absent in both VISTA mRNA high and low samples (data not shown). Although not investigated, such a lack of VISTA might reduce the likelihood of cancer development. Alternatively, subjects could acquire VISTA expression before or during malignant transformation, although in vitro differentiation did not drive VISTA expression in VISTA negative monocytes. A more in-depth analysis of expression patterns and persistence should be set-up in a longitudinal study in large population-based cohorts in order to gain insight into a potential role of VISTA in pathology.

In conclusion, the ectopic expression of VISTA drove the differentiation of macrophages towards a pro-tumoral M2-like phenotype in vitro and downregulated the key phagocytic signal SIRPα. Correspondingly, VISTA expressing macrophages more effectively phagocytosed cancer cells, with phagocytic activity correlating with endogenous VISTA expression in M2 macrophages only. Further, VISTA^ECD^ triggered phagocytosis of only cancer cells that have a VISTA binding partner, suggesting a ligand-dependent and -independent role of VISTA in phagocytosis of cancer cells. However, phagocytosis by VISTA-expressing macrophages was accompanied by increased secretion of anti-inflammatory cytokines and reduced MHC-mediated antigen presentation to T cells. Together, these findings suggest that VISTA is a negative innate immune checkpoint and position the exploration of VISTA antagonists to drive macrophage-mediated anticancer immunity.

## Materials and methods

### Antibodies and reagents

Fluorescent-conjugated mAbs used: CD14 (clone OFC14D), CD80 (clone MEM-233), CD86 (clone BU63), and HLA-ABC (clone W6/32) from Immunotools (Friesoythe, Germany); CD163 (clone GHI/61), VISTA (clone B7H5DS8) and HLA-A2 (clone BB7.2) from eBioscience (San Diego, CA, USA); CD206 (clone 15 − 2), CD11b (clone M1/70), SIRPα (clone 15–414), PDL-1 (clone MIH3), TIM-3 (clone F38-2E2), and SIGLEC-10 (clone 5G6) from BioLegend. Unconjugated mAbs used were VISTA (clone ABM5C53) from MyBioSource (San Diego, CA, USA) and HLA-ABC (clone W6/32) from BioLegend, and Rituximab (RTX, human IgG1 chimeric anti-CD20, Roche). The reagents used were: Incucyte® Cytolight Rapid Red Dye for Live-Cell Cytoplasmic Labeling (Cytolight Red) (Sartorius, Göttingen, Germany), Phorbol-12-myristate-13-acetate (PMA) (Thermo Scientific, London, UK), Lipopolysaccharide (LPS) (Merck, MO, USA), TGF-β (Peprotech, Thermo Scientific, London, UK), as well as Interleukin 4 (IL-4), Interleukin 10 (IL-10), Interleukin 13 (IL-13), interferon-γ (IFN-γ), granulocyte-macrophage colony-stimulating factor (GM-CSF), and macrophage-colony-stimulating factor (M-CSF) from Immunotools. Secretion of cytokines by macrophages was measured using appropriate ELISA kits (Immunotools).

### Cell lines and culture conditions

Human monocytic cell line THP-1, promyeloid cell line HL60 and DLBCL cell lines Daudi, SU-DHL-2, SU-DHL-4, SU-DHL-6, SU-DHL-10, U2932, SC1, OCI-LY3, RI1 were obtained from Deutsche Sammlung from Microorganism und Zellkulturen, (Braunschweig, Germany) or ATCC. Cell lines were cultured following the supplier’s recommendation at 37 °C in RPMI medium (Lonza, Biowhittaker BE12–604 F) supplemented with 10 or 20% fetal bovine serum (FBS, Gibco™ Fetal Bovine Serum, USA) in a humidified 5% CO2 environment. All cell lines underwent routine PCR testing for mycoplasma infections. THP-1 cells were differentiated to macrophages (M0 phenotype) with 100 ng/ml PMA for 48 h. THP-1 M0 macrophages were subsequently stimulated with human IFN-γ (20 ng/ml) and LPS (100 ng/ml) for 48 h to M1 phenotype, stimulated with human IL-4 (20 ng/ml) and IL-13 (20 ng/ml) for 72 h to M2a phenotype, or stimulated with human IL-10 (20 ng/ml) for 72 h to M2c phenotype.

### Primary samples and isolation of Immune cells

Peripheral blood samples from healthy donors were obtained as buffy coats from Sanquin, The Netherlands (under agreement number NVT0465.01). Peripheral blood mononuclear cells (PBMCs) from patients’ blood were isolated from anonymous waste material from newly diagnosed lymphoma patients. This study was carried out in The Netherlands in accordance with International Ethical and Professional Guidelines (the Declaration of Helsinki and the International Conference on Harmonization Guidelines for Good Clinical Practice). The use of anonymous waste material is regulated under the code for good clinical practice in the Netherlands. Informed consent was waived in accordance with Dutch regulations. Using Lymphoprep™ and density gradient centrifugation, peripheral blood mononuclear cells (PBMCs) were separated in accordance with the manufacturer’s instructions (STEMCELL Technologies, Vancouver, BC, Canada). Further, PBMCs (2.5 × 10^6^ cells/mL) were cultured in RPMI supplemented with 10% FBS, M-CSF or GM-CSF (50 ng/mL) for 7 days for differentiation to M0. The obtained M0 cells were exposed to LPS (100 ng/mL) and IFN- (20 ng/mL) for 1 day in order to produce M1-type macrophages, or IL-10 (50 ng/mL) and TGF-β (50 ng/mL) for 2 days in order to produce M2c-type macrophages.

### Lentiviral transduction

Lentiviruses were generated using transfer vector pRRL.SFFV.EGFP, VISTA or VISTA extracellular domain (ECD) by co-transfection of HEK293T cells with packaging construct psPAX2 (Addgene, #12,260) and Glycoprotein envelope plasmid pMD2.G (Addgene, #12,259) using Fugene (Promega, Madison, USA). HEK293T cells cultured in DMEM (-FCS + 1% pen/strep) were transiently transfected in T75 flasks. After 2 days, viral supernatant was harvested and filtered through a 0.45 μm filter, concentrated by ultracentrifugation and either used to transduce recipient cells directly (with 8 µg/mL polybrene to increase the infection efficiency) or stored in − 80 °C for future usage. Transduction efficiency was determined by GFP expression using flow cytometry. Stably transduced cells were sorted using a fluorescence-activated cell sorting system, Sony cell sorter SH800s (Sony Biotechnology, Tokyo, Japan).

### Single cell RNA sequence data analysis

Processed single cell RNA-Seq data was obtained from the Single Cell Portal (https://singlecell.broadinstitute.org/single_cell/study/SCP1288/tumor-and-immune-reprogramming-during-immunotherapy-in-advanced-renal-cell-carcinoma#study-summary). RNA-seq data of all identified tumor associated macrophages (TAMs) were used for analysis. Cells exhibiting zero VISTA expression were categorized as VISTA-negative, whereas those displaying any level of VISTA expression were classified as VISTA-positive. For differential expression analysis between VISTA-positive TAMs and VISTA-negative TAMs, a two-sided Wilcoxon rank-sum test with Bonferroni FDR correction was used.

### Quantitative real-time PCR

Total RNA was extracted from the cells following the instructions of the RNeasy Plus Mini Kit (Qiagen). 2 µg RNA was reverse transcribed using the iScriptTM cDNA Synthesis Kit from BioRad according to the manufacturer’s instructions. An equal amount of cDNA was amplified and quantified by using SSoAdvanced Universal SYBR® Green Supermix from BioRad on a Bio-Rad thermal cycler. The amplification programme consisted of 3 min at 95 °C, then 39 cycles of 5 s at 95 °C and 30 s at 58 °C, 3 s at 65 °C and finally 5 s at 95 °C. Melting curve was analyzed to determine primer specificity. 2^(-ΔCt) method was used for calculating with reference gene ribosomal protein L27 (RPL27). Primers used were: SIGLEC10_(forward AACGGAGCGTTTCTGGGAATCG/reverse TCTGAGTCCGTCTCTTCGGTAG), TIM 3 (HAVCR2)_(forward GACTCTAGCAGACAGTGGGATC/reverse GGTGGTAAGCATCCTTGGAAAGG), LILRB1_(forward CTCCCTATGAGTGGTCTCTACC/reverse CTGTTGTAGCCAGCATCAGAGC), SIGLEC5_(forward CTCACCTGTCAGATGAAACGCC/reverse CCGTTCCTGAAGATGGTGATGG), VSIG4_(forward GATGGCAACCAAGTCGTGAGAG/reverse CCTGGCATTGAAGGCTAATCCTC), SIRPA (forward TCTACAAGGTTGCATGAG/reverse GGTTCAGGTCTGCATATGTG), Vista_ (forward GCGGATGGACAGCAACATTC/reverse TGACTTTGGCCTCGGGTATC), PD-L1_ (forward TGGCATTTGCTGAACGCATTT/reverse AGTGCAGCCAGGTCTAATTGT), RPL27 (forward CCGGACGCAAAGCTGTCATCG/reverse CTTGCCCATGGCAGCTGTCAC).

### Staining

To detect expression of VISTA, monocytes from different donors (5 × 10^4^ cells) were pre-incubated with FcR blocking reagent (IVIG, Nanogam, Sanquin, The Netherlands) for 30 min, and followed by a 30-minute incubation at 4°C with anti-CD14-PE (Immunotools) and anti-VISTA-APC (eBioscience). Cells were washed twice with PBS and VISTA expression in CD14 positive cells was analyzed using flow cytometry (CytoFLEX, Beckman Coulter, Fullerton, CA, USA). Data was analyzed in the CytExpert Software (Beckman Coulter). A similar protocol was followed to stain with other antibodies.

### Detection of VISTA’s interacting partners

Cell lines (5 × 10^4 cells/tube) were treated with recombinant VISTA (SinoBiological) at 5 µg/mL (His tagged) for 1 h at 4 °C then washed with PBS twice. Anti-His tag-APC antibody (BioLegend) was added to the cells and incubated for another 1 h at 4 °C. Cells were washed twice with PBS and analyzed using flow cytometry.

### Phagocytosis assay

Prior to the experiment, THP-1 or donor-derived macrophages were harvested from plates using TrypLE Express (Life Technologies, Carlsbad, CA, USA) and cancer cell lines were fluorescently labeled with Incucyte Red according to manufacturer’s protocol. Cells were co-cultured at an Effector:Target (E:T) ratio of 1:1 and, where indicated, treated with 0.1 µg/mL RTX for 3-hour in RPMI 10% in a humidified 5% CO2 incubator at 37 °C. To identify primary macrophages after co-culture, the sample was stained with anti-CD11b-FITC followed by analysis using flow cytometry. Phagocytosis was quantified as the percentage of CD11b^+^ (or GFP^+^)/Incucyte red^+^ macrophages from the total CD11b^+^ (or GFP^+^). Confocal images were generated using Zeiss Cell Discoverer 7 with LSM900 confocal head. To get experimental replicates, assays were repeated with a minimum of three independent donors.

### HPV E7 antigen presentation assay

HPV-16 E7_11 − 20_ peptides (Peptides&Elephants) were used to pulse THP-1 derived macrophages for 2 h. The E7 antigen presentation ability of macrophages was analyzed by co-culturing macrophages with E7 TCR^+^ Jurkat NFAT-luminescence (firefly) reporter cells [38]. Activation of signal transduction in the TCR^+^ Jurkat reporter cells was measured by the production of luciferase after a 6-hour co-culture. In brief, Jurkat cells were collected from the wells into opaque white plates (Costar, Badhoevedorp, The Netherlands), whereupon 30 µL of Luciferase Assay Reagent Bio-Glo (Promega) was added to each well. After 15 min incubation, luminescence was measured using a luminescence reader (Synergy, BioTek, Winooski, VT, USA). The luciferase activity in Jurkat cells co-cultured with stimulated macrophages was expressed in relative luminescence units (RLUs), related to the luciferase activity of Jurkat cells co-cultured with non-stimulated macrophages.

### GFP-immunoprecipitation and mass spectrometry

Approximately 8 × 10^6^ cells were lysed in lysis buffer (150 mM NaCl, 50 mM Tris, pH 7.2, 20 nM EDTA, 20 nM EGTA, 1% NP-40 [Roche, 12,879,500], 0.1% SDS [Sigma, L6026]; containing Na3VO4 [Sigma, 450,243] and protease inhibitor cocktail [Sigmafast; Sigma Aldrich, S8820]), and protein concentration was determined using Bradford assay (Pierce™ Coomassie (Bradford) Protein Assay Kit, #23,200, Thermo Scientific, Waltham, MA, USA). Pre-clearance beads (25 µl per sample, Chromotek bmab-20) were washed 3x with ice cold wash buffer (10 mM Tris, 150 mM NaCl, 0.5 mM EDTA) and subsequently added to the lysate (2500 µg total protein in 400 µl lysis buffer). After 1-hour incubation at 4 °C, the lysate was harvested, and added to pre-washed GFP-beads (25 µl per sample, Chromotek gtma-20). After 1-hour incubation at 4 °C (on an end-over-end tumbler) the supernatant was removed and beads were washed thrice with ice cold wash buffer.

For HLA-ABC and VISTA immunoprecipitation, a total of 2500 µg of total protein in 400 µl of lysis buffer was incubated at 4 °C for 2 h with 5 µg of either anti-VISTA (MyBioSource) or anti-HLA-ABC (BioLegend) antibodies. Pre-clearance Dynabeads™ Protein G (ThermoFisher Scientific, 10003D) were washed 3x with ice cold wash buffer (10 mM Tris, 150 mM NaCl, 0.5 mM EDTA) and then added to the lysate. After overnight incubation at 4 °C with end-over-end tumbling, the supernatant was removed, and the beads were washed 3x with ice cold wash buffer. The beads were then resuspended in 100 µl of SDS-sample buffer (Laemmli buffer) containing β-ME and boiled for 10 min. The resulting supernatant was collected, and 20 µl was used for analysis by SDS-PAGE and immunoblotting, following previously described methods [39]. Further, 40 µl was used for mass spectrometry (Orbitrap-LC-MS), with LC-MS/MS and data analyses being performed essentially as previously described [40, 41].

### Molecular docking analysis

The molecular structures of both VISTA and HLA-ABC were obtained from UniProt (https://www.uniprot.org/). The investigation into the binding modes between VISTA and HLA-ABC was conducted through molecular docking using the HDOCK server [42–44]. The most optimal predicted binding model, as determined by the docking score provided by the HDOCK server, was subsequently visualized, analyzed, and mapped using PyMOL program version 2.4.0 (https://www.schrodinger.com/pymol). The docking scores were calculated by the knowledge-based iterative scoring function ITScorePP or ITScorePR. Confidence of interaction was quantified using a docking score-dependent confidence score that indicate likelihood of binding likeliness using the formula, Confidence_score = 1.0/[1.0 + e^0.02*(Docking_Score+150)^]. A confidence score above 0.7 indicates very likely occurrence of binding between the two molecules; a confidence score below 0.5 indicates the two molecules are unlikely to bind.

### Statistical analysis

Summary statistics are reported as mean and standard deviation (SD). Comparisons between 2 groups were performed using Student’s (or paired) Student’s *t* test. Comparisons among > 2 groups were performed using one-way ANOVA with *post hoc* intergroup comparisons. All statistical tests were performed using GraphPad Prism (GraphPad Prism; GraphPad Software, La Jolla, CA, USA). *p* values are indicated as: ***p < 0.001, **p < 0.01, and *p < 0.05.

### Electronic Supplementary Material

Below is the link to the electronic supplementary material.

Supplementary Material 1

## Data Availability

Data is provided within the manuscript or supplementary information files.
