# Supplementary material for: VISTA drives macrophages towards a pro-tumoral phenotype that promotes cancer cell phagocytosis yet down-regulates T cell responses
Source: Exp Hematol Oncol. 2024 Mar 29;13:35. doi: 10.1186/s40164-024-00501-x (PMC10979580; doi:10.1186/s40164-024-00501-x)
Supplement: Supplementary file 1 — Supplementary Material 1 [file 40164_2024_501_MOESM1_ESM.docx]

**Supplementary data**

**
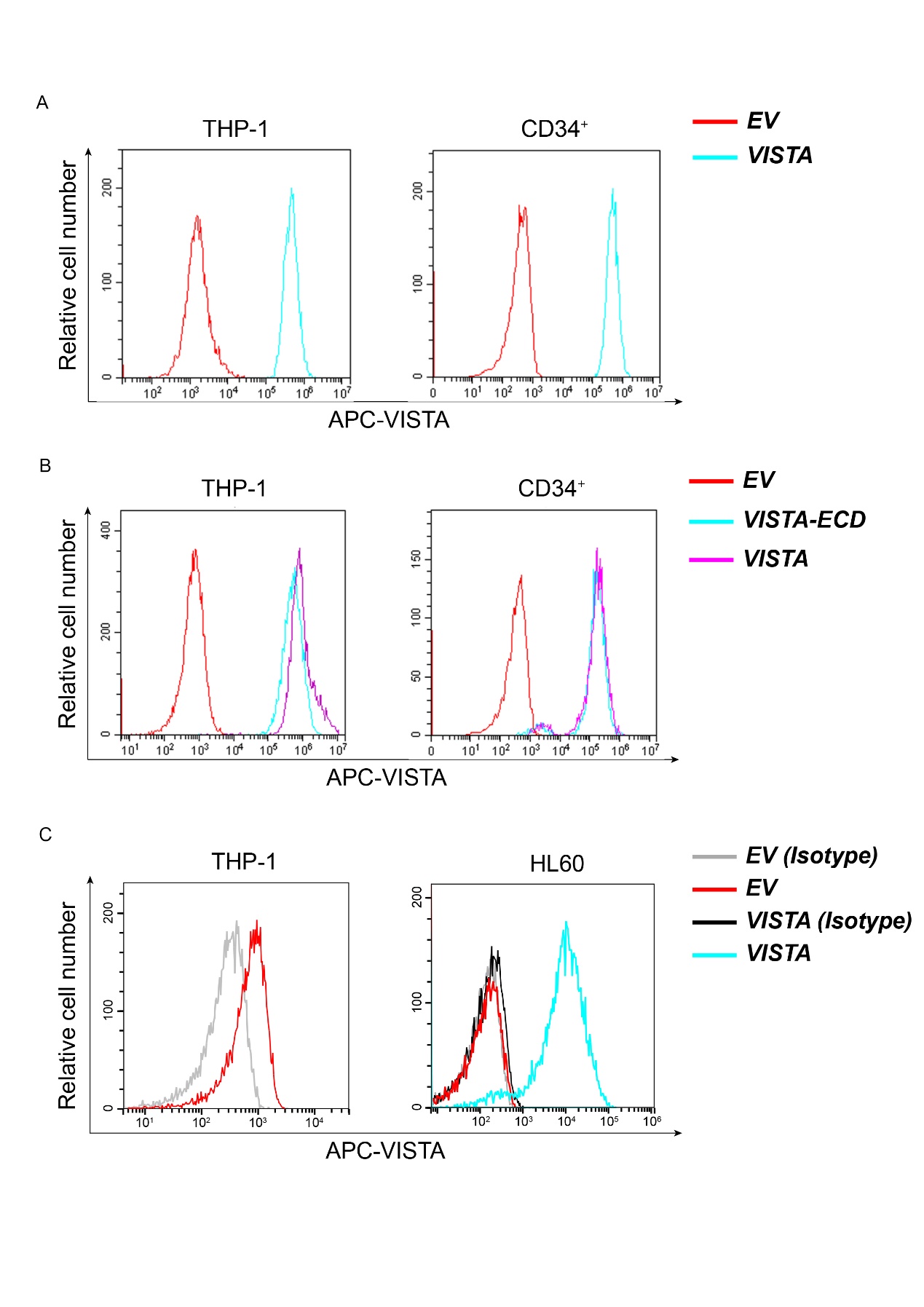
**

**Supplementary Figure 1**. **Expression of VISTA in different cell lines. A-B.** Flow cytometry plots illustrating the surface expression of VISTA on THP-1 cell line and CD34^+^ CB-derived macrophages under conditions of VISTA overexpression (A), or overexpression of VISTA and VISTA-ECD (B). **C.** Flow cytometry plot depicting the surface expression of VISTA on THP-1 cell line and HL60 cell line with VISTA overexpression.


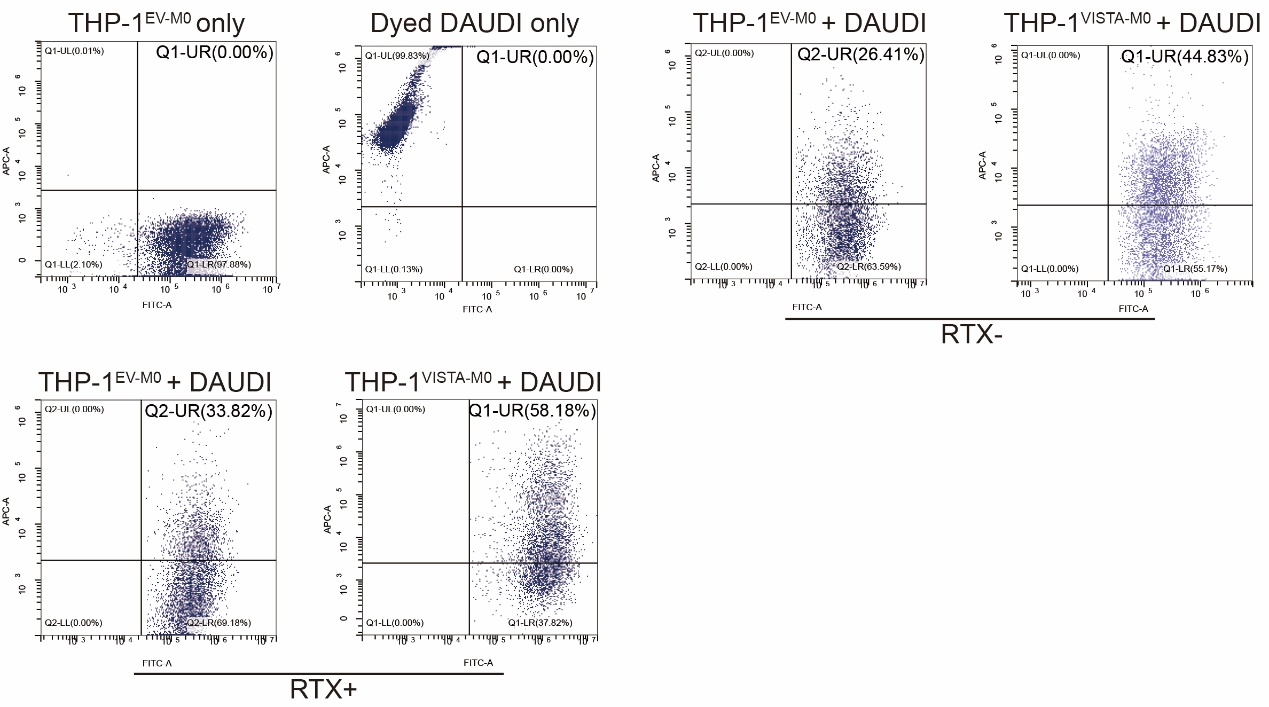


**Supplementary Figure 2. Representative FACS dot plots of phagocytosis assay.** Doublet formation of IncuCyte-red stained DAUDI cells and EV-GFP or VISTA-GFP transduced THP-1 cells. Doublets are detected as a double-positive FITC-APC signal (top right corner).


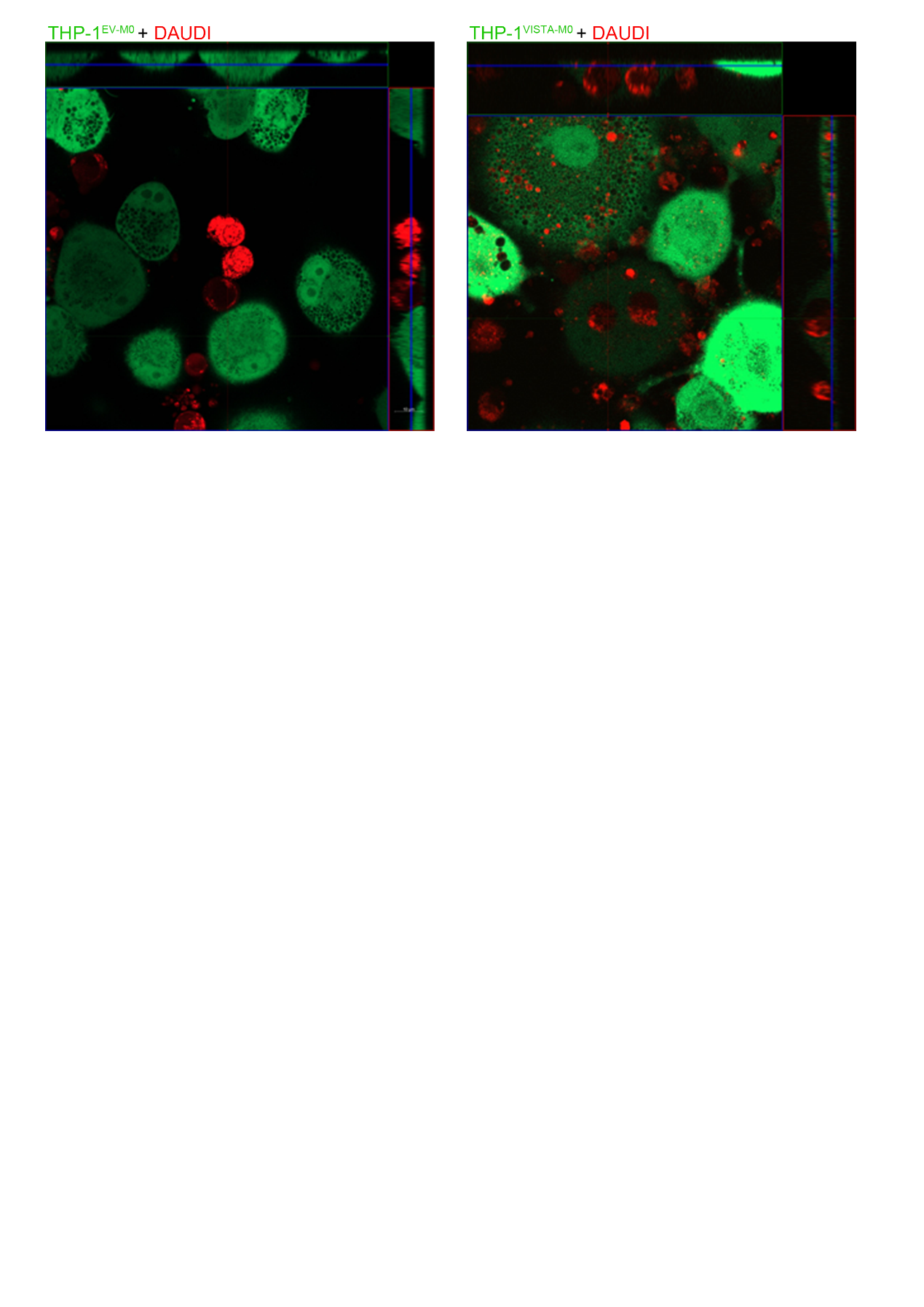


**Supplementary Figure 3. Representative confocal microscopy images of phagocytosis assay with THP-1 cells.** The pictures depicted THP-1 cell derived M0-like macrophage-mediated phagocytosis of DAUDI cells. DAUDI cells (red) were labelled with IncuCyte-red and macrophages (green) were with GFP tag.

**
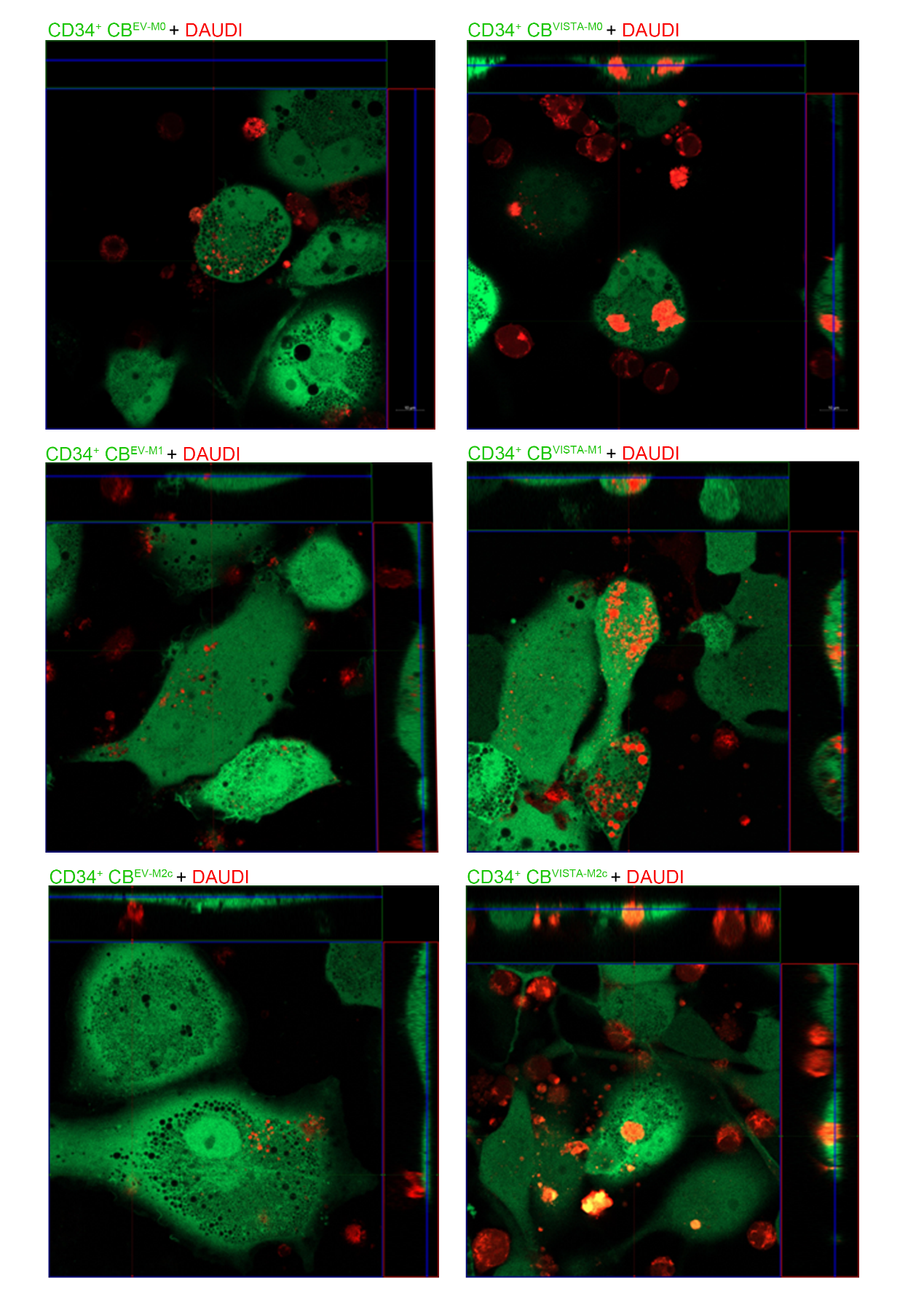
**

**Supplementary Figure 4. Representative confocal microscopy images of phagocytosis assay with CD34^+^ cord blood (CB) cells.** The pictures depicted CD34^+^ CB cell derived macrophage-mediated phagocytosis of DAUDI cells. DAUDI cells (red) were labelled with IncuCyte-red and macrophages (green) were with GFP tag.

**
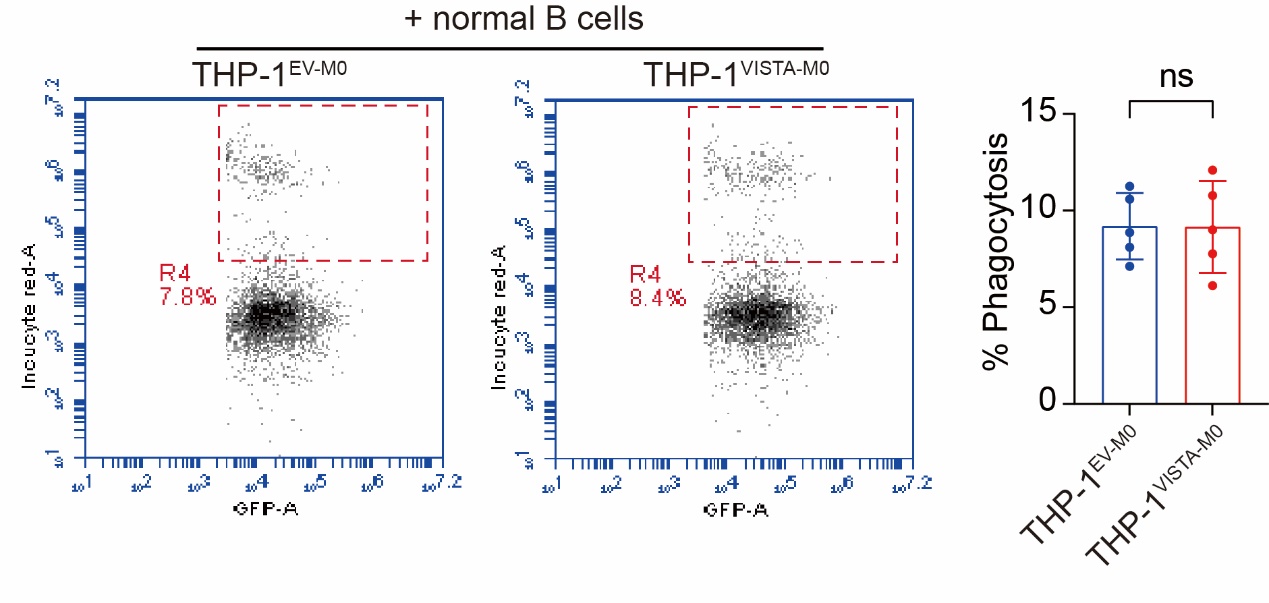
**

**Supplementary Figure 5. Phagocytosis of normal B cells by THP-1 cell derived M0-like macrophages.** Doublet formation of IncuCyte-red stained normal B cells and EV-GFP or VISTA-GFP transduced THP-1 cells. Doublets are detected as a double-positive FITC-APC signal (left panel, dashed square). Quantification of phagocytosis involving PBMC-derived normal B cells (n=5) by THP-1 derived M0-like macrophages (right panel). n.s., not significant by student’s t-test.

**
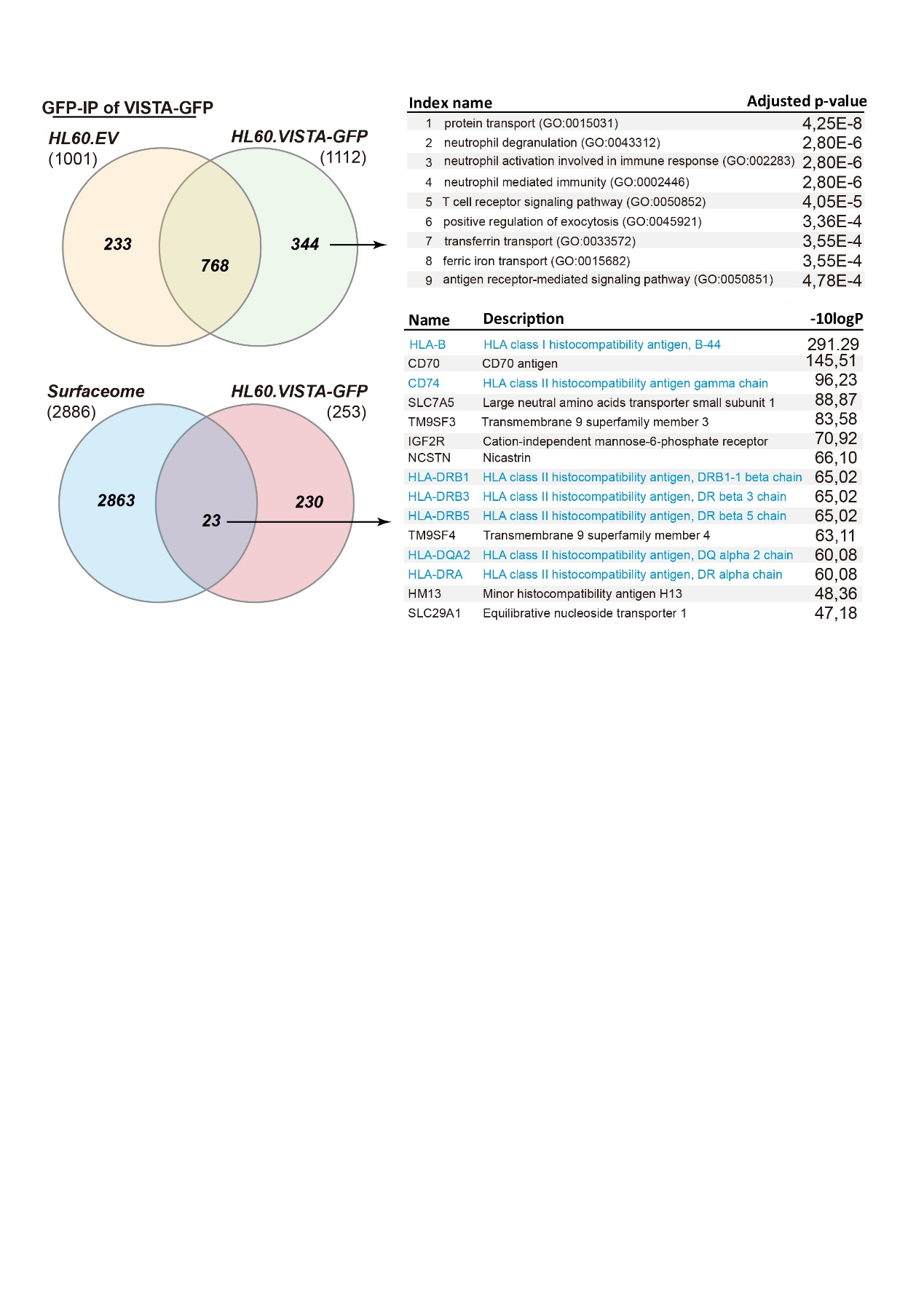
**

**Supplementary Figure 6**. **GFP-immunoprecipitation-mass spectrum (IP-MS) of HL60 expressing VISTA-GFP or EV-GFP.** A Venn diagram (upper panel) illustrating the specific hits associated with VISTA-GFP. Subsequent Gene Ontology (GO) term analysis was conducted on these hits. A Venn diagram (lower panel) highlighting transmembrane proteins exclusively interacting with VISTA-GFP, with HLA molecules highlighted in blue.

**Supplementary Table 1.** Summary of the top 10 docking models for VISTA and HLA-ABC

| **Interface residues** | model 1 | model 2 | model 3 | model 4 | model 5 | model 6 | model 7 | model 8 | model 9 | model 10 |
| --- | --- | --- | --- | --- | --- | --- | --- | --- | --- | --- |
| **Docking Score** | -252.82 | -252.11 | -241.12 | -238.83 | -237.31 | -230.78 | -227.95 | -225.15 | -219.26 | -218.79 |
| **Confidence Score** | 0.8866 | 0.8852 | 0.8609 | 0.8553 | 0.8515 | 0.8342 | 0.8262 | 0.8180 | 0.7998 | 0.7983 |
| **Ligand rmsd (Å)** | 604.75 | 607.61 | 611.89 | 587.23 | 588.20 | 623.57 | 588.78 | 598.62 | 616.65 | 578.63 |
